# 

**Published:** 1897-10

**Authors:** 


### CONTENTS.

ORIGINAL COMMUNICATIONS. PAGE
Regulating without Extraction *versus* Extraction for Regulating; Some Typical Comparative Results. By WM. SLOCUM DAVENPORT, D.D.S., Paris, France . 625
Electricity and Dentistry. By JAMES L. GETHINS, Boston, Mass. . . . . . . . 631
The Preparation of Dental Alloys and Cements. By LOUIS SHAW, M.D., Brooklyn, N. Y. . . . . . . . . . . . . . . . . . . . . . . . . . . . . 634
Clinical Report on Method of Operating. By DR. H. C. REGISTER, Philadelphia . 636
Gold-Shell Crowns One Hundred and Fifty Years Ago. By DR. WILLIAM H. TRUEMAN, Philadelphia . . . . . . . . . . . . . . . . . . . . . . . . 639
Some Features in Bridge-Work. By H. C. REGISTER, M.D. . . . . . . . . . . 641
The Physiology of Cataphoresis. By WESTON A. PRICE, Cleveland, Ohio . . . 647

ABSTRACTS AND TRANSLATIONS.
Gutta-Percha . . . . . . . . . . . . . . . . . . . . . . . . . . . . 651

REPORTS OF SOCIETY MEETINGS.
American Dental Association . . . . . . . . . . . . . . . . . . . . . 657
The New York Institute of Stomatology . . . . . . . . . . . . . . . . 660
Academy of Stomatology . . . . . . . . . . . . . . . . . . . . . . . 675
American Academy of Dental Science . . . . . . . . . . . . . . . . . 680
Massachusetts Dental Society . . . . . . . . . . . . . . . . . . . . 694

EDITORIAL.
The Entrance Examinations in Dental Colleges . . . . . . . . . . . . . 697

DOMESTIC CORRESPONDENCE.
Reply of Dr. Faught . . . . . . . . . . . . . . . . . . . . . . . . 700

CURRENT NEWS.
National Association of Dental Examiners . . . . . . . . . . . . . . . 700
Massachusetts Board of Registration in Dentistry . . . . . . . . . . . 701
Pennsylvania Examining Board . . . . . . . . . . . . . . . . . . . . 701

SELECTIONS.
Acetanilide as a Dressing . . . . . . . . . . . . . . . . . . . . . 701
Cocaine . . . . . . . . . . . . . . . . . . . . . . . . . . . . . . 702
Urotropine . . . . . . . . . . . . . . . . . . . . . . . . . . . . . 703

